# The effects of subacute exposure to a water-soluble cannabinol compound in male mice

**DOI:** 10.1186/s42238-022-00153-w

**Published:** 2022-07-27

**Authors:** Melissa M. Bailey, Mariah C. Emily Mills, Ashley E. Haas, Kelly Bailey, Robert C. Kaufmann

**Affiliations:** 1 Department of Biological Sciences, Emporia State University, 1 Kellogg Cir, Box 4050, Emporia, KS 66801 USA; 2 Research, Shaman Botanicals LLC, 2405 Southwest Blvd, Kansas City, MO 64108 USA

**Keywords:** Cannabinol, Acute toxicity, Mice, Water soluble

## Abstract

**Background:**

Cannabinol (CBN) is one of the many cannabinoids present in *Cannabis sativa* and has been explored as a potential treatment for sleeplessness. The purpose of this study was to determine the physiological and behavioral effects of subacute exposure to therapeutic and low pharmacological levels of a mechanically formed, stabilized water-soluble cannabinol nano-emulsion (CBNight™).

**Methods:**

Sixty-two male mice were randomly assigned to one of six treatment groups given CBNight™ at dosages designed to deliver 0mg (control) to 4 mg/kg of CBN daily via oral gavage for 14 days. In-cage behavior was observed at 30 minutes and at 2, 4, 8, and 16 hours after each dose. After 14 days, the mice were sacrificed and necropsied. Organs were weighed and inspected for gross abnormalities, and blood was collected via cardiac puncture for clinical chemistry.

**Results:**

No dosage-dependent adverse effects on behavior, body mass, or blood chemistry were observed, except that the highest doses of CBNight™ were associated with significantly lower eosinophil counts.

**Conclusions:**

The commercially available, water-soluble CBN compound employed in this study does not appear to cause adverse effects in mice; rather, it appears to be well tolerated at pharmacological levels. The findings of eosinopenia at higher doses of CBN and lack of hepatotoxicity at any dosage employed in this study have not been reported to date.

**Supplementary Information:**

The online version contains supplementary material available at 10.1186/s42238-022-00153-w.

## Background

Cannabinol (CBN) is one of the over 400 phytocannabinoids in the *C. sativa* plant. Its popularity is increasing as commercial preparations are becoming available to the public, particularly as an alternative sleep aid to over-the-counter (OTC) or prescription drugs. Cannabinoids including CBN have been shown in rodent studies to delay symptoms in amyotrophic lateral sclerosis (mice) (Weydt et al. 2005), to reduce myofascial pain (rats) (Wong and Cairns 2019), to act as an anticonvulsant (mice) (Karler et al. 1973) alternative treatment for mood disorders and anxiety (rodent and human) (Ferber et al. 2020), and to be a potential treatment for sleep disorders (rodent and human) (Murillo-Rodriguez et al. 2020; Kaufmann 2021a). The wide variety of applications for cannabinoids are possibly due in part to their activity at transient receptor potential vanilloid (TRPV) channels [reviewed in (Muller et al. 2018)] as well as cannabinoid (CB) receptors CB-1 and CB-2. The CBN compound, CBNight™ (CBNT), used in this study, was derived from hemp and is a mechanically formed, stabilized nano-emulsion of single lamellar, spherical vesicles with median and mode particle sizes of 55 nm. Some researchers reported that non-nano-treated CBN has a mild sedative effect at low doses (10–30 mg total dose or 0.17–0.5 mg/kg for a 60-kg human) (Musty et al. 1976)^,^while others have not (Hollister 1973; Hollister and Gillespie 1975). However, all researchers have reported that high levels of non-nano-treated CBN have no psychoactive effects in various rodent and human studies (Hollister 1973; Hollister and Gillespie 1975; Cohen and Weinstein 2018; Pertwee 2006). A recent survey of CBNT users found that nano-treated CBN (CBNT) at very low doses (1–4 mg total dose/night, 0.017–0.067 mg/kg/night for a 60-kg human) improved all aspects of sleep in humans, while, in some individuals, when the upper limit of the recommended dose (4 mg, 0.067 mg/kg for a 60-kg human) was taken, restlessness or extreme drowsiness could occur (Kaufmann 2021a). These findings seem to be unique for CBNT.

Unique findings with nano-treated cannabinoid compounds have been reported, and data from user surveys indicate that much lower doses of nano-treated CBD, CBN, and cannabigerol (CBG) are needed to produce similar perceived effects as higher doses of non-nano-treated cannabinoids (Kaufmann 2021a; Kaufmann 2021b; Kaufmann et al. 2021). Since cannabinoid metabolites are known to have biological effects (Ujvary and Hanus 2016; Huestis et al. 1992), it may be possible that these unique findings are due to differences in the relative percentage of metabolized cannabinoids to the native cannabinoid at the cellular level, although specific pharmacokinetic studies with CBNT have not been reported to date. If so, it is possible that nano-treated cannabinoids may be more, or less, toxic than non-nano-treated cannabinoids.

With the Food and Drug Administration (FDA) approval of Marinol and Epidiolex and the ever-growing popularity of cannabis and various phytocannabinoid nutritional supplements, there is an urgent need for further research, particularly on commercially available, non-FDA-approved supplement preparations (Bobst et al. 2020). Many of the previous evaluations of CBN toxicity (Chousidis et al. 2020; Colasanti et al. 1984; Takahashi and Karniol 1975; Thompson et al. 1973a; Thompson et al. 1973b) employed high pharmacological dosages of CBN preparations that were not commercially available (either laboratory-grade CBN or crude marijuana extract). The compound tested is currently commercially available and is being used by humans. With the general perception that these compounds are harmless, doses higher than those recommended may be ingested either accidentally or on purpose. Data on the potential for toxicity at doses higher than recommended are needed. The current study sought to assess the effects of a CBNT, a commercially available, water-soluble nanocompound marketed as a sleep aid and as an anxiolytic by using mice to examine its effects on the behavior and blood chemistry, and the potential for toxicity at a range encompassing recommended and excessive dosages.

## Methods

### Animals

Sixty-five male ICR mice 6–7 weeks old were purchased from the Envigo Laboratories (Indianapolis, IN). They were housed in gang housing (20 mice per cage, large cages) at the Emporia State University’s United States Department of Agriculture-approved animal facility and allowed to acclimate with handling only for cage changes (minimal handling) for 2 weeks. After acclimation, 60 mice were uniquely identified by ear punch and housed in cages of 3–4 mice within their treatment group so that all mice in a cage received the same treatment. Mice were allowed ad libitum access to Teklad Global Diet® 2018 (Envigo Laboratories, Madison, WI) and tap water. The room was maintained at 23 ± 3 °C with 30–40% humidity and a 12-h photoperiod. Two mice died and were replaced during the course of the study for a total of 62 animals involved. One mouse died late in the study and was not replaced. For details of animal deaths, including dosage groups and day of study, please see Table 1. All procedures performed on mice were in accordance with established guidelines set by the Emporia State University’s Institutional Animal Care and Use Committee that align with the National Research Council’s *Guide for Care and Use of Laboratory Animals*. The protocol was approved by the Emporia State University Institutional Animal Care and Use Committee (permit ESU-IACUC-21-002).

**Table 1:** Incidence of adverse clinical signs in mice dosed with CBNT

Clinical sign	Treatment group	Treatment day^**a**^	Number of affected mice	Total number of incidences^**b**^
Rough coat	Control (0 mg/kg/d)	9	1	1
	0.04 mg/kg/d CBN	4, 7, 8	5	5
	0.1 mg/kg/d CBN			
	0.4 mg/kg/d CBN	1, 2, 5–7, 13, 14	4	9
	1.0 mg/kg/d CBN	2, 4, 6, 7	8	9
	4.0 mg/kg/d CBN	9, 14	2	2
Ataxia	Control (0 mg/kg/d)			
	0.04 mg/kg/d CBN			
	0.1 mg/kg/d CBN			
	0.4 mg/kg/d CBN	6, 7	1	2
	1.0 mg/kg/d CBN			
	4.0 mg/kg/d CBN			
Rheum/eye irritation	Control (0 mg/kg/d)			
	0.04 mg/kg/d CBN			
	0.1 mg/kg/d CBN			
	0.4 mg/kg/d CBN	6, 7	1	2
	1.0 mg/kg/d CBN	1, 2	1	2
	4.0 mg/kg/d CBN			
Moribund sacrificed	Control (0 mg/kg/d)			
	0.04 mg/kg/d CBN			
	0.1 mg/kg/d CBN			
	0.4 mg/kg/d CBN			
	1.0 mg/kg/d CBN			
	4.0 mg/kg/d CBN	5	1	1
Found dead in age	Control (0 mg/kg/d)			
	0.04 mg/kg/d CBN	13*	1	1
	0.1 mg/kg/d CBN	4	1	1
	0.4 mg/kg/d CBN			
	1.0 mg/kg/d CBN			
	4.0 mg/kg/d CBN			

*Showed signs of rough coat 2 days prior to death. Not included in rough coat numbers since the mouse was later found dead. ^a^Treatment day refers to the days on which the sign was observed. ^b^Total number reflects the fact that some mice had symptoms that resolved and then returned. Each day of each mouse displaying the symptom is counted as 1 incident. *CBN*, cannabinol; *CBNT*, CBNight™; *d*, day; *kg*, kilogram; *mg*, milligram

### Test chemicals and collection materials

CBNight™ water-soluble dietary supplement (CBNT) containing 4 mg/ml CBN was supplied by the Shaman Botanicals (Kansas City, MO). This supplement is derived exclusively from hemp plants and is a mechanically formed, stabilized nano-emulsion. CBNT also contains a proprietary blend of polysorbate 60, water, and 2% or less of the following: sorbitan monostearate (food-grade emulsifiers), olive oil, ascorbic acid (preservative), α-bisabolol, terpineol, β-caryophyllene, linolool and α-pinene (terpines), and limonene (flavoring agent). CBNT treatment solutions were prepared daily prior to dosing by diluting CBNT with deionized (DI) water to achieve the desired concentration. Independent verification of CBN content is presented in Additional file 1.

Lavender-topped minitubes containing ethylenediaminetetraacetic acid (EDTA) anticoagulant (LTTs) and yellow-topped serum separator minitubes containing clot activator and gel (SSTs) were provided by IDEXX Laboratories (West Sacramento, CA).

### Treatments

The mice were randomly assigned to one of six treatment groups based on daily dosage: 0 mg/kg/d (vehicle control, DI water, *n* = 10); 0.04 mg/kg/d (*n* = 9); 0.1 mg/kg/d (*n* = 10) 0.4 mg/kg/d (*n* = 11), 1.0 mg/kg/d (*n* = 10), or 4.0 mg/kg/d (*n* = 11). All concentrations of the test compound were prepared either by diluting the stock (4 mg/ml) CBNT solution or by serial dilution using DI water as the diluent to achieve the appropriate test concentration. The vehicle control was DI water because even the most concentrated test solution was significantly diluted. All animals were weighed upon group assignment and again immediately prior to every dosing. Dosing occurred at 1400 h daily, 6 h into the light cycle. Test compounds or vehicle control was administered by oral gavage daily for 14 days. Administered solution volumes were adjusted based on individual animal weight to deliver the assigned dose. Animal equivalent treatment dosages were based on allometric scaling (Nair and Jacob 2016) and chosen to encompass and exceed the recommended dosage (0.017–0.067 mg/kg) for a 60-kg human.

### Data collection

#### Behavioral observation

At 30 min and at 2, 4, 8, and 16 h after each dosing, mice were observed in their home cages. General behaviors and clinical signs (gait, eye movement, coat condition, perioral/anal staining, and food/water consumption), activity level (from sleep to agitation/aggression), and unusual behaviors were noted. Food and water consumption were qualitatively, but not quantitatively, monitored. Any mice observed showing aggression leading to fighting or barbering of other mice were separated and housed singly for the remainder of the study. Researchers conducting behavioral observations were not blinded to the animal treatment groups; however, observation data were not reviewed by the researchers in totality until the completion of the study in an effort to minimize anticipatory observer bias.

#### Blood collection and necropsy

On day 15, mice were euthanized via CO_2_ overdose. Blood was collected from the heart chambers and aorta via cardiac puncture and placed into SSTs and LTTs (in order) and handled according to manufacturer instructions. The LTTs were refrigerated at 4 °C until shipping. The SSTs were allowed to sit at room temperature for a minimum of 20 min and then centrifuged at 500 × g for 10 min. After centrifugation, serum samples were drawn off and placed in separate collection tubes that were then frozen at −20 °C until shipping. All blood samples were stored and shipped individually and were not pooled. The SSTs and LTTs were packed according to laboratory recommendations and shipped overnight to IDEXX Laboratories (North Grafton, MA) for analysis. All blood testing was performed by IDEXX Laboratories.

A basic complete blood count (CBC) — i.e., white blood cell (WBC), red blood cell (RBC), hemoglobin (HGB), hematocrit (HCT), erythrocyte indices [mean corpuscular volume (MCV), mean corpuscular hemoglobin (MCH), and mean corpuscular hemoglobin concentration (MCHC)], leukocyte differential, and platelet estimates — was performed on whole blood samples. The CBC analyses were performed both by automated cell count and by manual differential cell count. A custom chemistry panel — i.e., alanine transaminase (ALT), aspartate transaminase (AST), albumin, total bilirubin, blood urea nitrogen (BUN), sodium, potassium, chloride, creatinine, globulin, glucose, and total protein — was performed on serum samples that had adequate volume. Alkaline phosphatase (ALP) was not measured in the blood samples. Samples requiring dilution by IDEXX Laboratories to achieve adequate volume were not analyzed for electrolytes.

After blood collection, the mice were necropsied. The lungs, heart, kidneys, and liver of each mouse were removed, examined for any gross abnormalities, and weighed. Left and right kidneys and lungs were examined and weighed separately. Organs were disposed of after examination and weighing.

### Data analyses

Morphometric and necropsy data were checked for homogeneity of variance using the Levene statistic; the null hypothesis was not rejected (*p* > 0.05). The data were then analyzed using a one-way analysis of variance (ANOVA), followed by a Tukey’s honestly significant difference post hoc test to determine specific differences using IBM SPSS, version 24.

Complete blood count and serum chemistry data were analyzed using SPSS PC + software. The homogeneity of variance between groups was checked by Bartlett’s test; the null hypothesis was not rejected. A one-way analysis was performed. Where the obtained result was positive, Duncan’s multiple range test was used to assess the significance of intergroup differences. Where significant heterogeneity was found, the normal distribution of data was examined by Kolmogorov-Smirnov test. In case of a nonnormal distribution, the nonparametric method of Kruskal-Wallis one-way ANOVA was used. If there was a positive result, the intergroup comparisons were performed using the Mann–Whitney test.

In all tests, a *p*-value of < 0.05 was considered statistically significant. All numerical data are reported as mean ± one standard deviation (SD).

## Results

### Treatment tolerance and necropsy data

Overall, the mice appeared to tolerate the treatment well. No clear dosage-dependent effect on the onset of signs, number of mice affected, or total number of signs of an incidence was observed (see Table 1). The most common clinical sign observed was rough coat, and it generally disappeared after 7 days of treatment. Ataxia and eye irritation/rheum were observed in a small number of mice, but these typically resolved in 2 days. One mouse in the 4.0 mg/kg/d dosage group became moribund and was sacrificed on day 5. Two mice in different treatment groups were not considered moribund prior to their discovery but were found dead at the 16-h post-dosing check. With the lack of dose-dependent relationship and differences in the timing of the deaths, it is difficult to draw firm conclusions about the effects of the treatment on morbidity and mortality. However, across all groups, 90% of the mice survived the duration of the study with only transient adverse signs and behaviors.

No significant differences in starting body mass or changes in mass were found among any of the groups (*p* > 0.05). No gross differences in food/water consumption were qualitatively observed. While food and water consumption were not quantitatively monitored on a per-animal basis, qualitative observations combined with a lack of significant difference in body mass within or among treatment groups suggest that there were no significant differences in consumption. There were no significant differences in lung, heart, liver, or kidney mass among the groups (see Table 2). There was an apparent reduction of liver mass in mice in the two highest dosage groups. This finding lacks statistical significance because of the use of repeated measure adjustments. None of the organs displayed gross abnormalities in any of the groups, and histopathology was not performed, as it was outside the scope of this project.

**Table 2:** Effects of CBNT on body and organ weight in mice

	Treatment group (mg/kg/d)					
	Vehicle control (0) *n* = 10	0.04 CBN *n* = 9	0.1 CBN *n* = 10	0.4 CBN *n* = 10	1.0 CBN *n* = 10	4.0 CBN *n* = 10
Initial body weight (g ± SD)	31.4 ± 2.9	31.2 ± 1.6	30.9 ± 2.5	31.8 ± 1.4	30.6 ± 2.2	29.9 ± 2.8
Weight change (g ± SD)	2.0 ± 1.6	3.2 ± 1.1	3.4 ± 1.6	2.3 ± 1.2	2.7 ± 1.5	1.9 ± 1.2
Average liver weight (g ± SD)	1.77 ± 0.14	1.73 ± 0.19	1.73 ± 0.22	1.71 ± 0.12	1.60 ± 0.17	1.52 ± 0.24
Average heart weight (g ± SD)	0.16 ± 0.03	0.16 ± 0.01	0.18 ± 0.03	0.17 ± 0.02	0.17 ± 0.02	0.16 ± 0.02
Average R lung weight (g ± SD)	0.17 ± 0.04	0.18 ± 0.02	0.18 ± 0.03	0.16 ± 0.03	0.19 ± 0.02	0.17 ± 0.04
Average L lung weight (g ± SD)	0.09 ± 0.02	0.09 ± 0.02	0.09 ± 0.02	0.08 ± 0.03	0.10 ± 0.02	0.09 ± 0.02
Average R kidney weight (g ± SD)	0.29 ± 0.02	0.30 ± 0.03	0.29 ± 0.04	0.29 ± 0.04	0.28 ± 0.03	0.27 ± 0.04
Average L kidney weight (g ± SD)	0.28 ± 0.02	0.29 ± 0.03	0.28 ± 0.04	0.28 ± 0.04	0.28 ± 0.02	0.27 ± 0.05

*CBN* cannabinol, *CBNT* CBNight™, *d* day, *kg* kilogram, *mg* milligram, *SD* standard deviation

### CBC data

A CBC was not performed on every blood sample; the missing samples (due to blood coagulation) were spread evenly between groups. There was no discernable relationship between administered dose and any of the CBC data points, except for eosinophil counts, where the average number and percentage of eosinophils were significantly lower in the three highest dose groups relative to the control group (see Tables 3 & 4).

**Table 3:** Effects of CBNT on complete blood count (CBC) in mice

	Treatment group (mg/kg/d)					
	Vehicle control (0)	0.04 CBN	0.1 CBN	0.4 CBN	1.0 CBN	4.0 CBN
**WBC (k/μL)**						
Mean ± SD	6.55 ± 2.05	6.62 ± 1.92	5.01 ± 2.10	5.533 ± 1.37	5.25 ± 1.22	4.97 ± 1.04
*n*	8	6	8	9	8	7
**RBC (M/μL)**						
Mean ± SD	9.85 ± 0.39	9.96 ± 0.29	10.20 ± 0.76	9.45 ± 1.14	10.24 ± 0.71	10.34 ± 0.39
*n*	7	6	8	9	8	7
**HGB (g/dL)**						
Mean ± SD	15.7 ± 0.8	15.9 ± 1.0	16.0 ± 0.9	15.2 ± 1.1	16.3 ± 1.0	16.1 ± 0.6
*n*	7	6	8	9	8	7
**HCT (%)**						
Mean ± SD	54 ± 4	56 ± 3	56 ± 2	52 ± 5	57 ± 4	56 ± 2
*n*	7	6	8	9	8	7
**MCV (fL)**						
Mean ± SD	54.9 ± 2.3	56.2 ± 1.2	55.0 ± 2.8	55.7 ± 2.2	55.3 ± 2.4	54.1 ± 1.8
*n*	7	6	8	9	8	7
**MCH (pg)**						
Mean ± SD	11.2 ± 7.7	10.7 ± 8.0	12.5 ± 6.6	14.6 ± 5.	14.2 ± 5.3	10.9 ± 7.5
*n*	10	9	10	10	9	10
**MCHC (g/dL)**						
Mean ± SD	29.2 ± 1.2	28.5 ± 0.6	28.6 ± 0.7	29.2 ± 2.2	28.9 ± 0.9	28.8 ± 0.9
*n*	7	6	8	9	8	7

*CBN* Cannabinol, *d* day, *HGB* Hemoglobin, *HCT* Hematocrit, *kg* kilogram, *MCH* mean corpuscular hemoglobin, *MCHC* Mean corpuscular hemoglobin concentration, *MCV* mean corpuscular volume, *mg* milligram, *RBC* Red blood cell, *SD* Standard deviation, *WBC* White blood cell

**Table 4:** Effect of CBNT on differential white blood cell counts in mice

	Treatment group (mg/kg/d)					
	Vehicle control (0)	0.04 CBN	0.1 CBN	0.4 CBN	1.0 CBN	4.0 CBN
*n*	8	6	8	9	8	7
**Lymphocytes**						
Mean ± SD	5791 ± 2014	6139 ± 1867	4530 ± 2047	5162 ± 1261	4036 ± 1853	4578 ± 1023
%	87.8 ± 7.2	92.3 ± 2.7	88.8 ± 7.3	93.3 ± 2.2	81.0 ± 32.8	91.9 ± 2.5
**Neutrophils**						
Mean ± SD	253 ± 1404	278 ± 176	328 ± 190	197 ± 136	222 ± 133	241 ± 53
%	3.9 ± 0.8	4.0 ± 2.2	8.0 ± 5.8	3.4 ± 2.0	4.4 ± 2.3	5.0 ± 1.5
**Band cells**						
Mean ± SD	0	0	0	0	0	0
%	0	0	0	0	0	0
**Monocytes**						
Mean ± SD	285 ± 192	110 ± 130	155 ± 172	153 ± 69	80 ± 45	147 ± 81
%	6.0 ± 7.3	1.6 ± 2.8	2.6 ± 2.8	2.6 ± 1.6	1.4 ± 1.0	2.1 ± 2.0
**Eosinophils**						
Mean ± SD	101 ± 97	89 ± 119	0^a^	21^a^ ± 34	25^a^ ± 27	6^a^ ± 15
%	1.6 ± 1.8	1.3 ± 1.6	0	0.3 ± 0.5	0.5 ± 0.5	0.1 ± 0.4
**Basophils**						
Mean ± SD	0	0	0	0	0.9 ± 2.5	0
%	0	0	0	0	0	0

^a^Differs significantly from control and 0.1 mg/kg/d CBN treatment groups. *CBN*, cannabinol; *CBNT*, CBNight™; *d*, day; *kg*, kilogram; *mg*, milligram; *SD*, standard deviation

### Blood chemistry

Blood chemistries were performed on all of the mice, except for 1 mouse in the 0.04 mg/kg/d group which died just prior to cardiac puncture. All the chemistry analyses were performed on the remaining mice, except for fifteen animals in whom sodium, chloride, and potassium levels were not performed due to a lack of adequate sample. Sixty-four percent (64%) of the blood draws had hemolysis which is known to raise AST, ALT, total bilirubin, protein, and potassium levels in proportion to the severity of the hemolysis. Correlation analysis of our data revealed that the severity of the hemolysis was correlated with AST, ALT, total bilirubin, protein, and potassium levels. No significant difference among groups was found in any of these blood chemistry components when values with hemolysis were eliminated (see Table 5). The number of samples and the severity of the hemolysis were very consistent between groups, such that when hemolyzed samples were included across all groups, no significant differences were found. Since the other blood chemistry components were not affected by degree of hemolysis, all available samples were analyzed, and no significant difference was found among groups (see Table 6).

**Table 5:** Effect of CBNT on blood chemistry in mouse blood samples without hemolysis

	Treatment group (mg/kg/d)											
	Vehicle control (0)		0.04 CBN		0.1 CBN		0.4 CBN		1.0 CBN		4.0 CBN	
	*n*	Mean ± SD	*n*	Mean ± SD	*n*	Mean ± SD	*n*	Mean ± SD	*n*	Mean ± SD	*n*	Mean ± SD
**AST** (IU/L)	4	74.50 ± 15.55	5	68.00 ± 27.90	4	98.25 ± 57.78	4	61.25 ± 29.44	2	89.50 ± 36.06	2	105.00 ± 66.47
**ALT** (IU/L)	4	27.50 ± 9.26	5	28.80 ± 11.95	4	48.75 ± 13.77	4	32.00 ± 13.83	2	31.00 ± 7.07	2	66.00 ± 56.57
**Total bilirubin** (mg/dL)	4	0.25 ± 0.10	5	0.20 ± 0	4	0.22 ± 0.05	4	0.25 ± 0.06	2	0.25 ± 0.07	2	0.25 ± 0.07
**Total protein** (g/dL)	4	5.63 ± 0.21	5	5.34 ± 0.27	4	5.52 ± 0.46	4	5.55 ± 0.26	2	5.55 ± 0.35	2	5.25 ± 0.07
**Potassium** (mmol/L)	3	10.23 ± 1.54	4	10.02 ± 0.55	2	9.25 ± 1.48	1	9.10	0		1	10.80

*ALT* Alanine transferase, *AST* Aspartate transaminase, *CBN* Cannabinol, *CBNT* CBNight™, *d* day, *dL* deciliter, *g* gram, *IU* International units, *kg* kilogram, *L* liter, *mg* milligram, *mmol* millimole, *n* number, *SD* standard deviation

**Table 6:** Effects of CBNT on blood chemistry in mouse blood samples with and without hemolysis

	Treatment group (mg/kg/d)											
	Vehicle control (0)		0.04 CBN		0.1 CBN		0.4 CBN		1.0 CBN		4.0 CBN	
	*n*	Mean ± SD	*n*	Mean ± SD	*n*	Mean ± SD	*n*	Mean ± SD	*n*	Mean ± SD	*n*	Mean ± SD
**Albumin** (g/dL)	10	3.07 ± 0.29	8	3.09 ± 0.26	10	3.19 ± 0.23	10	3.07 ± 0.11	10	3.06 ± 0.10	10	3.07 ± 0.26
**Globulin** (g/dL)	10	2.57 ± 0.15	8	2.46 ± 0.21	10	2.49 ± 0.18	10	2.50 ± 0.26	10	2.45 ± 0.14	10	2.48 ± 0.19
**BUN** (mg/dL)	10	19.80 ± 1.87	8	18.50 ± 2.00	10	18.80 ± 3.61	10	18.00 ± 1.76	10	18.00 ± 3.09	10	18.80 ± 2.70
**Creatinine** (mg/dL)	10	0.12 ± 0.08	8	0.062 ± 0.05	10	0.08 ± 0.08	10	0.07 ± 0.05	10	0.05 ± 0.05	10	0.08 ± 0.04
**Glucose** (mg/dL)	10	318.7 ± 57.21	8	298.00 ± 55.18	10	282.90 ± 54.17	10	315.6 ± 64.22	10	285.5 ± 33.50	10	332.70 ± 40.91
**Chloride** (mmol/L)	9	105.33 ± 2.52	6	105.83 ± 1.17	6	106.67 ± 1.86	7	106.14 ± 2.34	7	107.43 ± 1.27	8	106.25 ± 1.83
**Sodium** (mmol/L)	9	135.22 ± 47.57	6	152.83 ± 2.64	6	154.50 ± 2.95	7	151.43 ± 3.41	7	151.71 ± 1.98	8	152.62 ± 3.70

*CBN* Cannabinol, *CBNT* CBNight™, *d* day, *dL* deciliter, *g* gram, *kg* kilogram, *L* liter, *mg* milligram, *mmol* millimole, *n*, number, *SD* standard deviation

### Behavior observations

There was no clear dosage-dependent effect on behavior from CBN. Lethargy (defined as difficult to rouse and excessively sleepy when roused) was noted in three treatment groups, but these resolved by day 4 of treatment. Lethargy was generally observed at time points between 2 and 16 h post-dosing, whereas aggression and fighting (either resolved or requiring separation) were most commonly observed 30 min to 2 h post-dosing. At the 30-min observation, which occurred during the light cycle, mice became acclimated to dosing as the study progressed and tended to fall asleep by the 30-min observation within 5–7 days of the start of the study. The 2-h observation was conducted at 4:00 pm, which was still during the light cycle. Most mice, regardless of treatment group, became acclimated to dosing and handling and were asleep at this observation point. At the 8:00 and 10:00 pm observations (6-h and 8-h time points, respectively), mice were more active, and most were awake and engaged in normal activities such as eating, drinking, or grooming. The light cycle ended at 8:00 pm. At the 16-h time point (the next day’s 6:00 am observation), the majority of mice were asleep. Specific behaviors are summarized in Table 7.

**Table 7:** Behavioral observations in mice dosed with CBNT

Behavior	Treatment group	Treatment day^**a**^	Number of affected mice	Total number of incidences^**b**^
Lethargy	Control (0 mg/kg/d)			
	0.04 mg/kg/d CBN	3	2	2
	0.1 mg/kg/d CBN	1–3	3	5
	0.4 mg/kg/d CBN			
	1.0 mg/kg/d CBN			
	4.0 mg/kg/d CBN	2–4	1	3
Hypersensitivity/agitation when stimulated	Control (0 mg/kg/d)			
	0.04 mg/kg/d CBN			
	0.1 mg/kg/d CBN	3	1	1
	0.4 mg/kg/d CBN			
	1.0 mg/kg/d CBN	1.2	1	2
	4.0 mg/kg/d CBN	1–2	1	2
Transient aggression^c^	Control (0 mg/kg/d)	3.6	6	6
	0.04 mg/kg/d CBN			
	0.1 mg/kg/d CBN			
	0.4 mg/kg/d CBN			
	1.0 mg/kg/d CBN	4.5	3	3
	4.0 mg/kg/d CBN			
Aggression requiring separation^d^	Control (0 mg/kg/d)	4	2	2
	0.04 mg/kg/d CBN			
	0.1 mg/kg/d CBN			
	0.4 mg/kg/d CBN	3	4	4
	1.0 mg/kg/d CBN	2	2	2
	4.0 mg/kg/d CBN	1.10	2	2

*CBN*, cannabinol, *CBNT* CBNight™, *d* day, *kg* kilogram, *mg* milligram, *SD* standard deviation. ^a^Treatment day refers to the days on which the sign was observed. ^b^Total number reflects the fact that some mice had symptoms that resolved and then returned. Each day of each mouse displaying the symptom is counted as 1 incident. ^c^Transient aggression was defined as the observation of fighting between 2 mice that resolved with the observer tapping on the cage to distract the mice. ^d^Separation and subsequent housing were employed when mice continued fighting despite distraction efforts or if multiple incidences of fighting occurred during the observation period

## Discussion

The results of this study indicate that CBNT is a generally well-tolerated, nontoxic compound; this finding is consistent with a previous survey study on human subjects employing this compound (Kaufmann 2021a). The findings in this study are based upon a maximum dose that is approximately 5 times the maximum recommended human dose. Future studies that included dosages up to 25 mg/kg (the allometrically scaled animal equivalent of the dosage a 60-kg human would receive if consuming an entire standard bottle of CBNT containing 120 mg CBN in a single dose) would yield more information about possible toxicity with gross overconsumption.

Ocular irritation with discharge was noted but resolved within 2 days and was likely not related to the compound. There was no clear dose-dependent effect, as mice in the highest dosage group did not experience eye irritation. Additionally, incidences of ocular irritation were observed at different time points in the study. Conjunctival hyperemia after CBN administration in cats has been demonstrated more consistently, but the dosages were administered directly to the eye via mini-pump and not systemically (Colasanti et al. 1984), whereas oral administration was chosen in this study to mimic the recommended route of administration. Similarly, rough coat, ataxia, and morbidity/mortality did not occur in a dosage-dependent manner, and rough coat and ataxia were transient.

Our study did not find the significant effects of CBNT on body weight, weight gain, or organ weight that were observed by Thompson et al. (Thompson et al. 1973a) with chronic administration; however, the overall lack of body weight change was consistent with the results of their acute (7-day exposure) study (Thompson et al. 1973b). The CBN employed in both their acute and their chronic studies was administered in the form of crude marijuana extract (CME), of which 3.5% was CBN, in dosages that ranged from 48.3 or 115.5 mg CBN in a single injection in the acute study to 5.25–52.5 mg/kg/d CBN in the chronic study. The current study was designed to incorporate the minimum and maximum human dosage recommendations for CBNT specifically, and although the highest dosage tested (4 mg/kg) was lower than those employed by Thompson et al., it is approximately 5 times the maximum recommended human equivalent dosage for this product. Because the CBN was in the form of CME, specific effects observed cannot be contributed to CBN alone. The apparent decrease in liver mass observed in the two highest dosage groups in this study was not statistically significant and was not associated with any changes in liver tests suggesting that, if CBNT has any effect on the liver, it is not associated with significant hepatoxicity. However, additional studies examining this topic, including a longer duration of dosing, higher dosages, and histopathology to determine whether significant hepatocellular and biliary cell degeneration and fibrosis are occurring, would help to resolve this question.

Agitation/aggression (whether resolved or requiring separation) and lethargy were observed in our study, and these observations are consistent with studies in rats and zebra fish (Chousidis et al. 2020; Takahashi and Karniol 1975; Thompson et al. 1973a). The agitation/aggression observed was more likely to be due to handling by experimenters than the compound, since incidences of both were also observed in control animals and resolved within the first week of the study as the mice appeared to become accustomed to handling.

CBN has been thought to be stimulatory or sedating, depending on dose; however, this study found no dosage-dependent effect. In extremely high doses (80 mg/kg), CBN has been shown to delay the onset of sleep and rapid eye movement (REM) sleep (Colasanti et al. 1984). This effect has been observed in human studies as well, where low dosages (1 mg total dose, 0.017 mg/kg for a 60-kg human) of CBN uniformly caused relaxation and sleepiness, whereas higher dosages (3–4 mg total dose, 0.05–0.067 mg/kg for a 60-kg human) did not cause sleepiness in some individuals but which resolved quickly by lowering the dose (Kaufmann 2021a). Conflicting reports on the potential psychoactive properties of CBN exist (Karniol et al. 1975; Perez-Reyes et al. 1973), but comparisons between these studies are difficult because the route of administration and the dosages were significantly different.

Significant eosinopenia was observed (*p* < 0.05) in the 3 highest dosage groups compared to control animals; this finding has not been reported in the literature. However, modulation of the immune system by cannabinoids is well known. CB2 receptors are found throughout the immune system, and binding of CBN has been shown to suppress lymphoproliferation via inhibition of adenylyl cyclase and nuclear factor-κB in thymocytes (Herring et al. 1998; Herring and Kaminski 1999), indicating that CBN has anti-inflammatory and immunosuppressive effects. Previous studies have indicated that CBN has the ability to significantly reduce levels of interleukins (IL), including IL-2, IL-4, IL-5, and IL-13 mRNA, to attenuate excess mucus production, and to reduce serum immunoglobulin E (IgE) levels (Jan et al. 2003). Research is ongoing on the potential of cannabinoid products to serve as primary or secondary treatments for diseases that are rooted in or are worsened by inflammation (Suryavanshi et al. 2020). No significant effect on lymphocytes, monocytes, neutrophils, or basophils was observed in this study.

This study found no signs of hepatotoxicity of CBNT. Although the blood chemistry sample size was very limited for some of the blood chemistry parameters due to sample hemolysis, there were no differences between groups for any of the liver chemistries (ALT, AST, and bilirubin) when the hemolytic samples were excluded or included. ALP was not measured in this study because previous literature indicates that non-synthetic cannabinoid compounds do not generally affect ALP without significant ALT or AST elevation when administered alone, and several studies have reported either no (Thompson et al. 1973a) or mild ALP elevation (Borini et al. 2004) or that altered liver enzyme levels were likely due to co-administration of other drugs (Chesney et al. 2020). Additionally, as the focus of this study was on discovering potential toxicity, ALT and AST were chosen as endpoints for this study because they specifically indicate hepatocellular degeneration, whereas elevated ALP levels are more likely to indicate enzyme induction, but inclusion of ALP in future studies is planned. Clinically meaningful ALT elevation has been shown typically with repeated high dosages (> 1000 mg/d) of cannabinoids, particularly CBD (Chesney et al. 2020; Ewing et al. 2019a; Watkins et al. 2021), but in some cases, elevation was attributed to high levels of CBD used in conjunction with another drug. The potential for hepatotoxicity has been a concern with delta-9-THC and CBD, although conflicting reports in the literature have made the risk posed by these cannabinoids difficult to determine with certainty. Activation of the endocannabinoid system in the liver via CB1 receptors has been thought to play a role in fibrosis and nonalcoholic and alcoholic fatty liver disease (Tam et al. 2011). Some studies have indicated that chronic marijuana usage, especially in conjunction with chronic alcohol consumption, increases the incidence of hepatomegaly and alters liver aminotransferase levels (Borini et al. 2004; Kew et al. 1969). There have been some indications that high levels of CBD, alone or in conjunction with other hepatically metabolized drugs such as acetaminophen, may also result in hepatotoxicity (Ewing et al. 2019a; Ewing et al. 2019b). However, there has been overwhelming evidence to the contrary, with clinical studies and reviews indicating a lack of significant hepatotoxicity (Stohs 2020; Goyal et al. 2018) and some even showing hepato-protective roles for cannabinoids in fatty liver disease (Adejumo et al. 2018) and reducing the hepatotoxicity of other co-administered compounds (Uzma and Ambreen 2019).

The administration of cannabinoid compounds with lipids or in lipid formulations allows for preferential absorption into the intestinal lymphatics avoiding the first-pass liver metabolism. This has been shown to result in a higher percentage of the unmetabolized cannabinoid being distributed to the body (Zgair et al. 2017). Nanotechnology has allowed for the development of cannabinoid and other phytochemical products with higher bioavailability than non-nano-treated products (Atsmon et al. 2018; Nakano et al. 2019; Du et al. 2022; Wang et al. 2008; Lin et al. 2011; Gunasekaran et al. 2014; Wu et al. 2008). Because serum protein binding of cannabinoids is extremely rapid and nano-treated cannabinoid droplets entering the bloodstream in the intestine are so small, serum protein binding of the cannabinoids prior to entering the liver may help to further avoid first-pass metabolism (Fanali et al. 2011; Grotenhermen 2003). While specific pharmacokinetic studies have not been reported on CBNT, it is possible that this nano-emulsion also displays increased bioavailability compared to non-nano-treated formulations. Despite this possible increase in bioavailability, none of our results indicate that CBNT causes toxicity, even at dosages that are higher than recommended.

The results of this study, even with a relatively small sample size, indicate that CBNT is well tolerated, even at supra-recommended dosages, and that subacute exposure does not appear to cause lasting undesirable side effects or clinical signs of toxicity. Because CBNT is a blend of CBN and terpenes, this effect cannot be ascribed solely to CBN. The significance and specific mechanism of the eosinopenia observed requires further research; however, our results are encouraging and align with the results of others. Additional research to discern the specific role of terpenes and CBN play on the effects observed is an area of interest, as is further investigation of high dosages of CBNT on liver morphology and function. As nanotechnology continues to make compounds, such as the one used in this study, more highly bioavailable and more accessible to the public, laboratories must continue to research the potential benefits and risks associated with them. This need will increase as the interest in cannabinoid compounds will likely only grow stronger.

## Conclusions

The results of this study indicate that a water-soluble nano-preparation of CBN does not cause significant toxicity at low pharmacological or at non-pharmacological dosages in mice. No significant dosage-dependent adverse effects on behavior, body mass, organ mass, or blood chemistry were observed, except that the highest doses of CBN were associated with significantly lower eosinophil counts. Additionally, no lasting adverse behavioral effects were observed that can be directly linked to the compound. Our findings align well with what has been observed in humans using CBNight™ as a sleep aid (Kaufmann 2021a). The significance and underlying mechanisms of the eosinopenia observed are an area of interest, and further research is planned to explore whether this effect is observed in other animal species, including humans. Chronic exposure studies with CBNight™, as these compounds are often used daily for long periods of time, are of interest, as are studies aimed at elucidating the relationship, mechanism, and specific role of the terpenes and CBN in CBNT. As cannabinoid compounds continue to rise in popularity for recreational and medicinal usage and novel, highly bioavailable compounds continue to be produced, and continued research evaluating the safety and efficacy of them remains relevant and vital.

## Supplementary Information

**Additional file 1.** Certificate of Analysis CBNT.

## Data Availability

The dataset generated during the current study are available from the corresponding author on reasonable request.

## Abbreviations

ALT: Alanine transaminase
ANOVA: Analysis of variance
AST: Aspartate transaminase
BUN: Blood urea nitrogen
CBC: Complete blood count
CBD: Cannabidiol
CBG: Cannabigerol
CBN: Cannabinol
CBNT: CBNight™
CME: Crude marijuana extract
DI: Deionized
EDTA: Ethylenediaminetetraacetic acid
FDA: Food and Drug Administration
HCT: Hematocrit
HGB: Hemoglobin
Ig: Immunoglobulin
LTT: Lavender-topped minitube
MCH: Mean corpuscular hemoglobin
MCHC: Mean corpuscular hemoglobin concentration
MCV: Mean corpuscular volume
RBC: Red blood cell
SST: Serum separator minitube
THC: Tetrahydrocannabinol
TRPV: Transient receptor potential vanilloid
WBC: White blood cell

## References

[CR1] Adejumo AC, Ajayi TO, Adegbala OM, Adejumo KL, Alliu S, Akinjero AM (2018). Cannabis use is associated with reduced prevalence of progressive stages of alcoholic liver disease. Liver Int..

[CR2] Atsmon J, Cherniakov I, Izgelov D, Hoffman A, Domb AJ, Deutsch L (2018). PTL401, a new formulation based on pro-nano dispersion technology, improves oral cannabinoids bioavailability in healthy volunteers. J Pharm Sci..

[CR3] Bobst S, Ryan K, Plunkett LM, Willett KL (2020). ToxPoint: toxicology studies on delta9-tetrahydrocannabinol and cannabidiol-containing products available to consumers are lacking. Toxicol Sci..

[CR4] Borini P, Guimaraes RC, Borini SB (2004). Possible hepatotoxicity of chronic marijuana usage. Sao Paulo Med J..

[CR5] Chesney E, Oliver D, Green A, Sovi S, Wilson J, Englund A (2020). Adverse effects of cannabidiol: a systematic review and meta-analysis of randomized clinical trials. Neuropsychopharmacology.

[CR6] Chousidis I, Chatzimitakos T, Leonardos D, Filiou MD, Stalikas CD, Leonardos ID (2020). Cannabinol in the spotlight: toxicometabolomic study and behavioral analysis of zebrafish embryos exposed to the unknown cannabinoid. Chemosphere..

[CR7] Cohen K, Weinstein AM (2018). Synthetic and non-synthetic cannabinoid drugs and their adverse effects-a review from public health prospective. Front Public Health..

[CR8] Colasanti BK, Craig CR, Allara RD (1984). Intraocular pressure, ocular toxicity and neurotoxicity after administration of cannabinol or cannabigerol. Exp Eye Res..

[CR9] Du X, Hu M, Liu G, Qi B, Zhou S, Lu K (2022). Development and evaluation of delivery systems for quercetin: a comparative study between coarse emulsion, nano-emulsion, high internal phase emulsion, and emulsion gel. J Food Eng.

[CR10] Ewing LE, McGill MR, Yee EU, Quick CM, Skinner CM, Kennon-McGill S (2019). Paradoxical patterns of sinusoidal obstruction syndrome-like liver injury in aged female CD-1 mice triggered by cannabidiol-rich cannabis extract and acetaminophen co-administration. Molecules..

[CR11] Ewing LE, Skinner CM, Quick CM, Kennon-McGill S, McGill MR, Walker LA (2019). Hepatotoxicity of a cannabidiol-rich cannabis extract in the mouse model. Molecules..

[CR12] Fanali G, Cao Y, Ascenzi P, Trezza V, Rubino T, Parolaro D (2011). Binding of delta9-tetrahydrocannabinol and diazepam to human serum albumin. IUBMB Life..

[CR13] Ferber SG, Namdar D, Hen-Shoval D, Eger G, Koltai H, Shoval G (2020). The “entourage effect”: terpenes coupled with cannabinoids for the treatment of mood disorders and anxiety disorders. Curr Neuropharmacol..

[CR14] Goyal H, Rahman MR, Perisetti A, Shah N, Chhabra R (2018). Cannabis in liver disorders: a friend or a foe?. Eur J Gastroenterol Hepatol..

[CR15] Grotenhermen F (2003). Pharmacokinetics and pharmacodynamics of cannabinoids. Clin Pharmacokinet..

[CR16] Gunasekaran T, Haile T, Nigusse T, Dhanaraju MD (2014). Nanotechnology: an effective tool for enhancing bioavailability and bioactivity of phytomedicine. Asian Pac J Trop Biomed..

[CR17] Herring AC, Kaminski NE (1999). Cannabinol-mediated inhibition of nuclear factor-kappaB, cAMP response element-binding protein, and interleukin-2 secretion by activated thymocytes. J Pharmacol Exp Ther..

[CR18] Herring AC, Koh WS, Kaminski NE (1998). Inhibition of the cyclic AMP signaling cascade and nuclear factor binding to CRE and kappaB elements by cannabinol, a minimally CNS-active cannabinoid. Biochem Pharmacol..

[CR19] Hollister LE (1973). Cannabidiol and cannabinol in man. Experientia..

[CR20] Hollister LE, Gillespie H (1975). Interactions in man of delta-9-tetrahydrocannabinol. II. Cannabinol and cannabidiol. Clin Pharmacol Ther..

[CR21] Huestis MA, Henningfield JE, Cone EJ, Blood cannabinoids. I. (1992). Absorption of THC and formation of 11-OH-THC and THCCOOH during and after smoking marijuana. J Anal Toxicol..

[CR22] Jan TR, Farraj AK, Harkema JR, Kaminski NE (2003). Attenuation of the ovalbumin-induced allergic airway response by cannabinoid treatment in A/J mice. Toxicol Appl Pharmacol..

[CR23] Karler R, Cely W, Turkanis SA (1973). The anticonvulsant activity of cannabidiol and cannabinol. Life Sci..

[CR24] Karniol IG, Shirakawa I, Takahashi RN, Knobel E, Musty RE (1975). Effects of delta9-tetrahydrocannabinol and cannabinol in man. Pharmacology..

[CR25] Kaufmann R (2021). Use of a water-soluble form of cannabinol for the treatment of sleeplessness. Int J Complement Alt Med..

[CR26] Kaufmann R (2021). Nano-processed CBG/CBD effect on pain, adult attention deficit hyperactive disorder, irritable bowel syndrome and chronic fatigue syndrome. Int J Complement Alt Med..

[CR27] Kaufmann R, Aqua K, Lombard J, Lee M. Observed impact of long-term consumption of oral cannabidiol on liver function in healthy adults. Cannabis Cannabinoid Res. 2021; Dec 16 Online ahead of print. https://www.liebertpub.com/doi/10.1089/can.2021.0114. 10.1089/can.2021.0114 PMC9940803 34918948

[CR28] Kew MC, Bersohn I, Siew S (1969). Possible hepatotoxicity of cannabis. Lancet..

[CR29] Lin W, Hong J-L, Shen G, Wu RT, Wang Y, Huang M-T (2011). Pharmacokinetics of dietary cancer chemopreventive compound dibenzoylmethane in rats and the impact of nanoemulsion and genetic knockout of Nrf2 on its disposition. Biopharmaceut Drug Disposition..

[CR30] Muller C, Morales P, Reggio PH (2018). Cannabinoid ligands targeting TRP channels. Front Mol Neurosci..

[CR31] Murillo-Rodriguez E, Budde H, Veras AB, Rocha NB, Telles-Correia D, Monteiro D (2020). The endocannabinoid system may modulate sleep disorders in aging. Curr Neuropharmacol..

[CR32] Musty REKI, Shirikawa I, Takahashi RN, Knobel E, Braude MC, Szara S (1976). Interactions of delta-9-tetrahydrocannabinol and cannabinol in man. The Pharmacology of Marihuana.

[CR33] Nair AB, Jacob S (2016). A simple practice guide for dose conversion between animals and human. J Basic Clin Pharm..

[CR34] Nakano Y, Tajima M, Sugiyama E, Sato VH, Sato H (2019). Development of a novel nano-emulsion formulation to improve intestinal absorption of cannabidiol. Med Cannabis Cannabinoids..

[CR35] Perez-Reyes M, Timmons MC, Davis KH, Wall EMA (1973). comparison of the pharmacological activity in man of intravenously administered delta9-tetrahydrocannabinol, cannabinol, and cannabidiol. Experientia..

[CR36] Pertwee RG (2006). Cannabinoid pharmacology: the first 66 years. Br J Pharmacol..

[CR37] Stohs SJRSD (2020). Is cannabidiol hepatotoxic or hepatoprotective: a review. Toxicol Res Appl.

[CR38] Suryavanshi SV, Kovalchuk I, Kovalchuk O (2020). Cannabinoids as key regulators of inflammasome signaling: a current perspective. Front Immunol..

[CR39] Takahashi RN, Karniol IG (1975). Pharmacological interaction between cannabinol and Δ9=tetrahydrocannabinol. Psychopharmacologia..

[CR40] Tam JLJ, Mukhopadhyay B, Cinar R, Godlewski G, Kunos G (2011). Endocannabinoids in liver disease. Hepatology..

[CR41] Thompson GR, Mason MM, Rosenkrantz H, Braude MC (1973). Chronic oral toxicity of cannabinoids in rats. Toxicol Appl Pharmacol..

[CR42] Thompson GR, Rosenkrantz H, Schaeppi UH, Braude MC (1973). Comparison of acute oral toxicity of cannabinoids in rats, dogs and monkeys. Toxicol Appl Pharmacol..

[CR43] Ujvary I, Hanus L (2016). Human metabolites of cannabidiol: a review on their formation, biological activity, and relevance in therapy. Cannabis Cannabinoid Res..

[CR44] Uzma RAMI, Ambreen A (2019). Evaluation of the heaptoprotectice effect of aqueous extract of *Cannabis sativa* (marijuana) agaist diclofenac sodium induced hepatotoxicity. J Toxicol Pharmaceut Sci..

[CR45] Wang X, Jiang Y, Wang YW, Huang MT, Ho CT, Huang Q (2008). Enhancing anti-inflammation activity of curcumin through O/W nanoemulsions. Food Chem..

[CR46] Watkins PB, Church RJ, Li J, Knappertz V (2021). Cannabidiol and abnormal liver chemistries in healthy adults: results of a phase I clinical trial. Clin Pharmacol Ther..

[CR47] Weydt P, Hong S, Witting A, Moller T, Stella N, Kliot M (2005). Cannabinol delays symptom onset in SOD1 (G93A) transgenic mice without affecting survival. Amyotroph Lateral Scler Other Motor Neuron Disord..

[CR48] Wong H, Cairns BE (2019). Cannabidiol, cannabinol and their combinations act as peripheral analgesics in a rat model of myofascial pain. Arch Oral Biol..

[CR49] Wu TH, Yen FL, Lin LT, Tsai TR, Lin CC, Cham TM (2008). Preparation, physicochemical characterization, and antioxidant effects of quercetin nanoparticles. Int J Pharm..

[CR50] Zgair A, Lee JB, Wong JCM, Taha DA, Aram J, Di Virgilio D (2017). Oral administration of cannabis with lipids leads to high levels of cannabinoids in the intestinal lymphatic system and prominent immunomodulation. Sci Rep..

