# Supplementary material for: The effects of subacute exposure to a water-soluble cannabinol compound in male mice
Source: J Cannabis Res. 2022 Jul 27;4:44. doi: 10.1186/s42238-022-00153-w (PMC9327251; doi:10.1186/s42238-022-00153-w)
Supplement: Supplementary file 1 — Additional file 1. Certificate of Analysis CBNT. [file 42238_2022_153_MOESM1_ESM.pdf]

CERTIFICATE OF ANALYSIS
ISO/IEC 17025:2017 ACCREDITATION #103104

Order #: 47906
Order Name: Regular CBN Water Soluble Natural
Batch#: 10004232
Received: 02/05/2020
Completed: 02/06/2020

American Shaman
2405 Southwest Blvd
Kansas City Missouri, 64108
(913) 486-9889
info@cbdamericanshaman.com

Sample

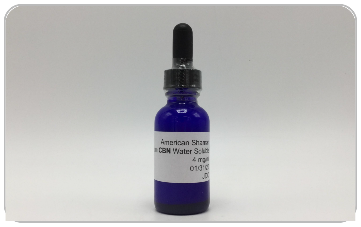

N/D<br>D9-THC	N/D<br>Total CBD
125.4 mg<br>Cannabinoids per bottle	N/D mg<br>CBD per bottle

1 bottle = 30 ml per bottle x density (0.945) x Cannabinoid concentration

Cannabinoids Test

SHIMADZU INTEGRATED UPLC-PDA
GSL SOP 400 **PREPARED:** 02/05/2020 17:02:49 **UPLOADED:** 02/06/2020 12:00:18

Cannabinoids	LOQ	weight(%)	mg/g	mg/bottle
D9-THC	10 PPM	N/D	N/D	N/D
THCA	10 PPM	N/D	N/D	N/D
CBD	10 PPM	N/D	N/D	N/D
CBDA	20 PPM	N/D	N/D	N/D
CBDV	20 PPM	N/D	N/D	N/D
CBC	10 PPM	N/D	N/D	N/D
CBN	10 PPM	**0.442%**	**4.422**	**125.4**
CBG	10 PPM	N/D	N/D	N/D
CBGA	20 PPM	N/D	N/D	N/D
D8-THC	10 PPM	N/D	N/D	N/D
THCV	10 PPM	N/D	N/D	N/D
TOTAL D9-THC		**N/D**	**N/D**	**N/D**
TOTAL CBD*		**N/D**	**N/D**	**N/D**
TOTAL CANNABINOIDS		**0.442%**	**4.422**	**125.4**

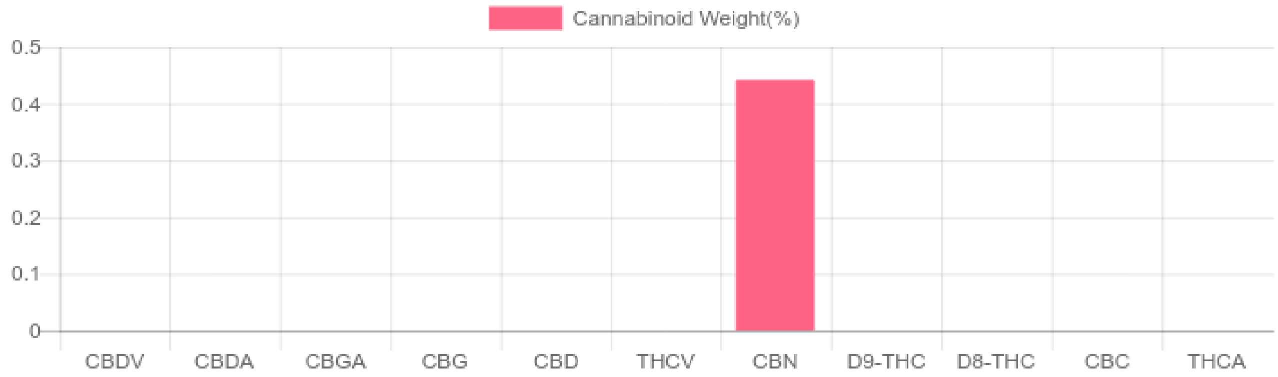

Reporting Limit 10 ppm
*Total CBD = CBD + CBDA x 0.877
N/D - Not Detected, B/LOQ - Below Limit of Quantification

Dr. Andrew Hall, Ph.D.,Chief Scientific Officer

Ben Witten, MS, MT., Lab Director

Green Scientific Labs
info@greenscientificlabs.com
1-833 TEST CBD

Green Scientific Labs uses its best efforts to deliver high quality results and to verify that the data contained therein are based on sound scientific judgment and levels listed are guidelines only and all data was reported based on standard laboratory procedures and deviations. However Green Scientific Labs makes no warranties or claims to that effect and further shall not be liable for any damage or misrepresentation that may result from the use or misuse of the data contained herein in any way. Further, Green Scientific Labs makes no claims regarding representations of the analyzed sample to the larger batch from which it was taken. Data and information in this report are intended solely for the individual(s) for whom samples were submitted and as part of our strict confidentiality policy, Green Scientific Labs can only discuss results with the original client of record.
